# Naturally Occurring PCSK9 Inhibitors: An Updated Review

**DOI:** 10.3390/molecules30173582

**Published:** 2025-09-02

**Authors:** Jungmoo Huh, Hyunwoo Kim

**Affiliations:** 1 Research Institute of Pharmaceutical Sciences, College of Pharmacy, Seoul National University, Seoul 08826, Republic of Korea; goodhjm112@snu.ac.kr; 2 College of Pharmacy and Integrated Research Institute for Drug Development, Dongguk University-Seoul, Goyang 10326, Republic of Korea

**Keywords:** PCSK9, natural products, LDL, hypercholesterolemia, cardiovascular disease

## Abstract

Proprotein convertase subtilisin/kexin type 9 (PCSK9) is a key modulator of low-density lipoprotein cholesterol (LDL-C) levels and emerged as an attractive therapeutic target for the treatment of hypercholesterolemia and cardiovascular diseases. Although statins and ezetimibe have been widely used to manage these disorders, concerns regarding side effects and high costs have driven ongoing efforts to search for alternative therapeutic candidates. To date, several classes of PCSK9 inhibitors, including monoclonal antibodies, oligonucleotides, proteins, and peptides, have been approved or are under clinical trials. In this review, we summarize 57 newly identified compounds derived from natural products showing inhibitory effects against PCSK9 reported between 2020 and April 2025. These compounds were isolated from 18 plants species and belong to various structural classes, including isoprenoids, flavonoids, alkaloids, and phenolic derivatives.

## 1. Introduction

**Figure 1:**
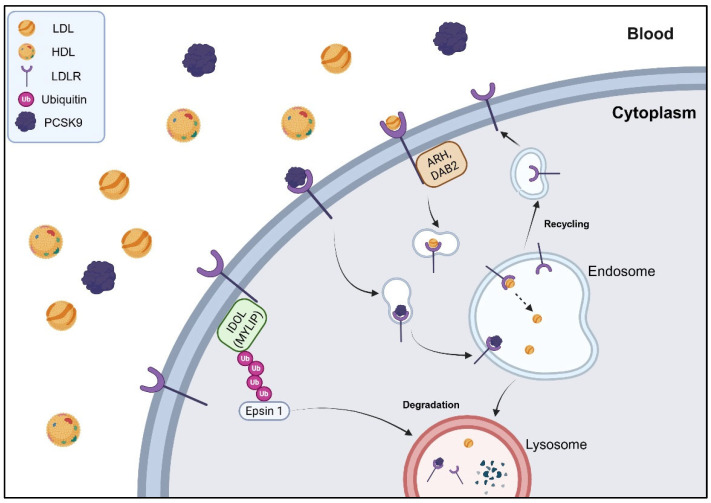
The role of PCSK9 in LDLR regulation. The figure was created with Biorender.com, with permission.

Cholesterol plays a vital role as a key component of cell membranes in the human body, contributing to their structure, permeability, and fluidity [1]. In addition, cholesterol also acts as a precursor for the biosynthesis of steroid and sex hormones, bile acids, vitamin D, and lipoproteins [2,3]. Cholesterol is transported through the body in the form of lipoproteins, which circulate in the bloodstream [4]. These lipoproteins are classified into two types, low-density lipoproteins (LDLs) and high-density lipoproteins (HDLs) [2,4]. When cholesterol levels become imbalanced, particularly when they are elevated, the formation of plaque in blood vessels is facilitated, raising the risk of cardiovascular diseases, atherosclerosis, and hypercholesterolemia [2,4,5,6]. To reduce the risk of these diseases and manage elevated cholesterol levels, physicians have prescribed statin compounds and ezetimibe [2,7,8,9]. However, due to individual medical histories, genetic factors, and side effects of statin treatment, the need for alternative therapeutic agents has become increasingly important [10,11,12,13,14]. The proprotein convertase subtilisin/kexin type 9 (PCSK9) was discovered in 2003 [15], and it is mainly produced in the liver in humans [15]. PCSK9 plays a key role in cholesterol homeostasis by regulating the levels of LDL receptors (LDLRs) on hepatocyte surfaces [16,17]. PCSK9 binds to LDLRs and promotes their degradation in lysosomes, thereby reducing the ability to remove LDL cholesterol (LDL-C) from the bloodstream [16,18,19] (Figure 1). As a result, elevated PCSK9 activity leads to increased circulating LDL-C levels, contributing to a higher risk of atherosclerosis and cardiovascular disease [16,20]. Given its pivotal role in lipid metabolism, PCSK9 has emerged as an attractive therapeutic target for managing hypercholesterolemia, liver diseases, and associated cardiovascular conditions.

To date, several PCSK9 inhibitors have either been approved or are currently under clinical trials. Most of these inhibitors are monoclonal antibodies (alirocumab [21], evolocumab [22], bococizumab [23], LY3015014 [24], ongericimab/JS002 [25], tafolecimab/IBI306 [26], ebronucimab/AK102 [27], and recaticimab [28]), while others include proteins (LIB003 [29]), peptides (MK-0616 [30], NNC0385-0434 [31], and PCSK9 adnectin [32]), oligonucleotides (Inclisiran [33]), and small molecules (AZD0780 [34] and BMS-962476 [35]). Additionally, several natural-product-derived compounds such as berberine [16,17], ginkgolide B [36], lupin [17,36], polyphenols (quercetin [17], resveratrol [17,36], EGCG [17], and curcumin [36]), lycopene [17,36], etc., have been reported to exhibit inhibitory effects on PCSK9.

In this review, we summarize natural-product-derived compounds reported from 2020 to April 2025 that exhibit inhibitory effects on PCSK9 secretion. Although several reviews on PCSK9 inhibitors derived from natural products have been reported, they typically focused on specific disorders, such as atherosclerosis [37], hypercholesterolemia [36], and cardiovascular diseases [16,38,39], or reviewed the mechanisms associated with these diseases [16,36,40]. Some previous reviews on natural products have provided updates on well-known compounds such as berberine [16,17], resveratrol [17,41,42], and curcumin [17,42]. In contrast, this review compiles and categorizes a broad range of compounds that have been newly isolated from natural sources and confirmed to exhibit inhibitory effects against PCSK9.

## 2. Results and Discussion

A total of 350 compounds have been isolated from natural products and evaluated for their inhibitory activity against PCSK9 secretion from January 2020 to date. Among them are 57 newly identified compounds with PCSK9 inhibitory effects that were not covered in a previous review published in 2020 [17]. A diverse range of compound classes, including isoprenoids, flavonoids, alkaloids, and phenolic derivatives, were isolated from 18 different plant species.

### 2.1. The Naturally Occurring PCSK9 Inhibitors Reported in 2020

In 2020, Li et al. [43] isolated a cucurbitane-type triterpenoid derivative, 23,24-dihydrocucurbitacin B (**1**), from the ethanolic extract of *Trichosanthes cucumeroides* roots. Their work on HepG2 cells revealed that 23,24-dihydrocucurbitacin B (**1**) upregulated low-density lipoprotein receptor (LDLR) protein expression, leading to a dose-dependent elevation of 1,1′-dioctadecyl-3,3,3′,3′-tetramethylindocarbocyanine perchlorate-labeled low-density lipoprotein (DiI-LDL) uptake. Furthermore, the researchers investigated the relationship between PCSK9 and LDLRs, finding that 23,24-dihydrocucurbitacin B (**1**) reduced PCSK9 protein levels while increasing LDLR mRNA levels. Mechanistically, compound **1** modulates the transcription of PCSK9 and LDLRs via HNF1 and SRE1 motifs, respectively, by altering the nuclear levels of HNF-1α and SREBP2. These results were confirmed by dose-dependent Western blot and qPCR analyses using concentrations of 1, 2, 5, and 10 μM. The study also included in vivo experiments with HFD-fed hamsters, which showed that 23,24-dihydrocucurbitacin B (**1**) decreased total cholesterol, triglyceride, and LDL-C levels, while regulating LDLR and PCSK9 expression in the liver. A Western blot analysis of the liver tissues confirmed decreased PCSK9 and HNF-1α levels and increased LDLR and SREBP2 levels, along with the upregulation of SREBP2 target genes (HMGCR and HMGCS1).

A study by Zhang et al. [44] reported the isolation of 7 new and 20 known cucurbitacins from *T. cucumeroides*. To establish structure–activity relationships (SARs), the research team also synthesized 22 derivatives. A total of 47 cucurbitacins were then evaluated at 5 μM for their LDL uptake activity in HepG2 cells. The results showed that hexanorisocucurbitacin D (**2**) and isocucurbitacin D (**3**) exhibited greater LDL uptake than the positive control, nagilactone B. The subsequent SAR analysis demonstrated that a 2-oxo-3α-hydroxy A-ring is crucial for activity. It was also noted that while modifications at C-2, C-3, or C-16 had little impact, the presence of a carbonyl group at C-7 or a methoxy group at C-24 could enhance LDL uptake. Further Western blot analysis revealed that both hexanorisocucurbitacin D (**2**) and isocucurbitacin D (**3**) increased LDLR protein levels at concentrations of 5, 10, and 20 μM and decreased PCSK9 protein levels at 10 and 20 μM.

A study by Pel et al. [45] reported the purification of 31 compounds, including a stilbene dimer, flavonoids, and phenolic acids, from the methanolic extract of aerial parts of *Chromolaena odorata* to assess their inhibitory activity on PCSK9 expression in HepG2 cells. Prior to this isolation, the research team had already confirmed the inhibitory effects of the crude extract and its subsequent fractions. Among the isolated compounds, (+)-8b-*epi*-ampelopsin A (**4**), 5,6,7,4′-tetramethoxyflavanone (**5**), 5,6,7,3′,4′-pentamethoxyflavanone (**6**), acacetin (**7**), and uridine (**8**) showed inhibitory activity against PCSK9 mRNA expression, with IC_50_ values of 20.6, 21.4, 31.7, 15.0, and 13.7 μM, respectively.

**Figure 2:**
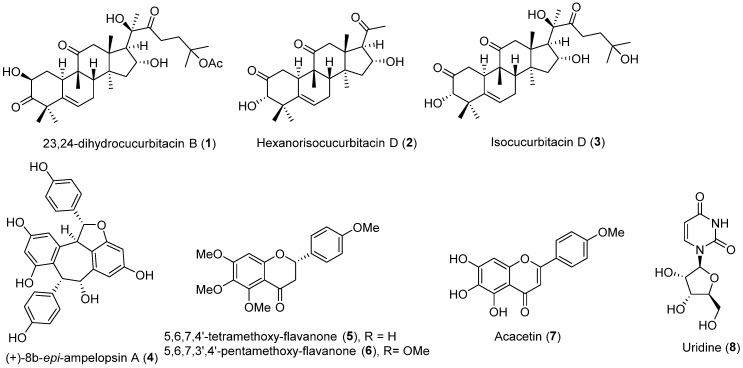
Chemical structures of the naturally occurring PCSK9 inhibitors reported in 2020.

Based on its potency and available quantity, 5,6,7,4′-tetramethoxyflavanone (**5**) was selected for deeper investigation. Further evaluation using Western blot analysis showed that treatment with 5,6,7,4′-tetramethoxyflavanone (**5**) at 10, 20, and 40 μM suppressed PCSK9 protein expression while elevating LDLR protein levels at 10 and 20 μM. These results suggest that the decrease in PCSK9 production led to enhanced LDLR protein recycling rather than its lysosomal degradation. Given that transcription factors like SREBP and HNF-1α are known to regulate PCSK9 [46], the authors investigated this pathway. Based on the observed suppression of HNF-1α mRNA expression, they concluded that 5,6,7,4′-tetramethoxyflavanone (**5**) might regulate PCSK9 via the HNF-1α pathway. The chemical structures of the PCSK9 inhibitors reported in 2020 are depicted in Figure 2, and the summarized information is presented in Table 1.

### 2.2. The Naturally Occurring PCSK9 Inhibitors Reported in 2021

In 2021, a study by Nhoek et al. [47] described the isolation of 7 new sesquiterpenes and 12 known compounds from the aerial parts of *Salvia plebeian*. All isolated compounds were tested at 50 μM for inhibitory effects on PCSK9 mRNA expression in HepG2 cells. Among them, plebeic acid A (**9**), (1*S*,5*S*,8*S*,10*R*)-1-acetoxy-8-methoxy-2-oxoeudesman-3,7(11)-dien-8,12-olide (**10**), and eudebeiolide B (**11**) showed significant inhibitory activity, with IC_50_ values of 24.4, 25.2, and 27.8 μM, respectively. The two most potent compounds, plebeic acid A (**9**) and eudebeiolide B (**11**), also moderately upregulated LDLR mRNA expression. A subsequent Western blot analysis revealed that both compounds slightly reduced PCSK9 protein and increased LDLR protein levels at 10 and 50 μM, though the effects were less potent than those of the positive control, berberine. The investigation also included a PCSK9-LDLR binding assay, but no isolates showed activity.

Also in 2021, Weng et al. [48] reported 20 dammarane-type triterpenoidal saponins from *Gynostema pentaphyllum*. To assess lipid-lowering activity, the authors selected eight compounds for PCSK9 inhibition experiments in HepG2 cells. The results showed that the gypenosides LXXXIX (**12**), XC (**13**), and XCI (**14**), and the ginsenoside Rg5 (**15**) significantly inhibited simvastatin-induced PCSK9 expression at 20 μM. The gypenosides LXXXIX (**12**) and XC (**13**) and the ginsenoside R_g5_ (**15**), in particular, demonstrated potent suppression of PCSK9 expression even at a lower concentration of 10 μM.

Kim et al. [49] isolated and reported 4 new prenylated flavonoid glycosides along with 18 known compounds from the dried aerial parts of *Epimedium koreanum*. They tested all isolated compounds and their inhibitory effects of PCSK9 mRNA expression and modulation of LDLR mRNA expression in HepG2 cells. Ten compounds, icariside I (**16**), ikarisoside A (**17**), icariin (**18**), anhydroicaritin 3-*O*-β-d-fucopyranosyl(1→2)-rhamnopyranoside-7-*O*-β-d-glucoside (**19**), korepimedoside A (**20**), epimedokoreanoside I (**21**), korepimeoside C (**22**), epimedin L (**23**), caohuoside B (**24**), and epimedoicarisoside A (**25**), showed inhibitory effects against PCSK9 expression at 10 μM. Notably, only ikarisoside A (**17**) elevated LDLR mRNA expression, suggesting that this compounds has potential for use as a cholesterol-lowering drug.

Woo et al. [50] reported five new selaginellin derivatives along with one known selaginellin from *Selaginella tamariscina* roots and rhizophores. Among the isolated compounds, selaginpulvilin U (**26**) showed the highest upregulation of LDLR-related genes at 50 μM in human HepG2 cells. The authors further evaluated selaginpulvilin U (**26**) for its effects on LDLR transcript and protein levels in a dose-dependent manner and found that LDLR expression was regulated via SREBPs.

**Figure 3:**
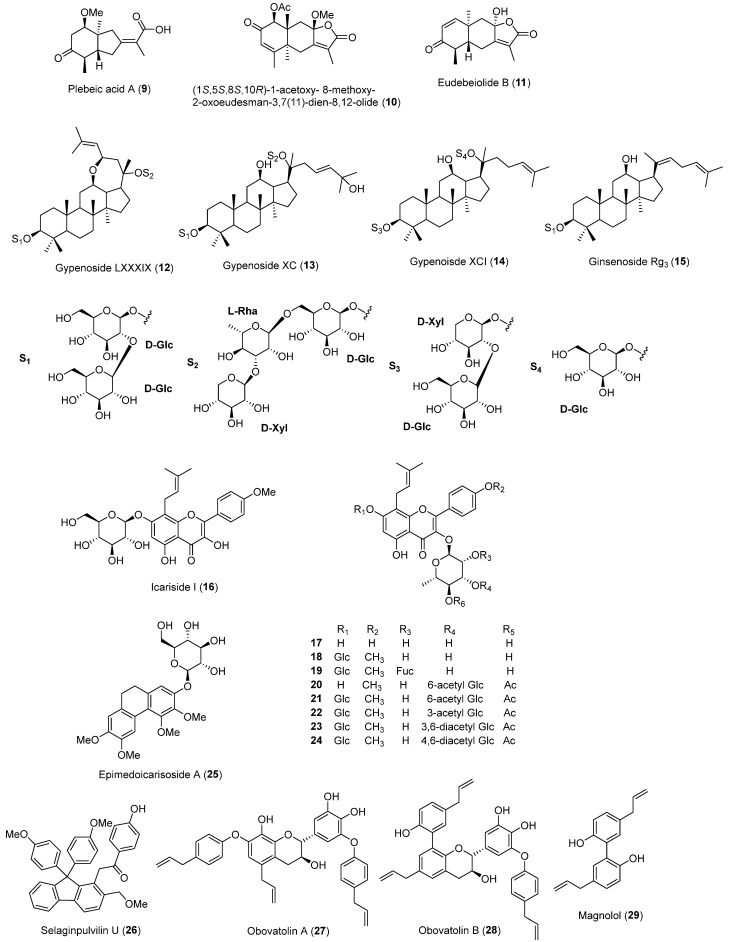
Chemical structures of the naturally occurring PCSK9 inhibitors reported in 2021.

Ahn et al. [51] isolated two lignan dimers, obovatalins A (**27**) and B (**28**), along with magnolol (**29**) from the dried bark of *Magnolia obovate*. The authors investigated the effect of these compounds on PCSK9 expression levels, and the results showed that compounds **27**–**29** exhibited inhibitory effects on PCSK9 expression in HepG2 cells, with IC_50_ values of 12.0, 45.4, and 22.9 μM, respectively. In a further investigation, obovatalin A (**27**) notably reduced PCSK9 protein levels and increased LDLR expression. The chemical structures of the PCSK9 inhibitors reported in 2021 are shown in Figure 3, and the summarized information is presented in Table 2.

### 2.3. The Naturally Occurring PCSK9 Inhibitors Reported in 2022

In 2022, Huang et al. [52] isolated and reported dammarane-type saponins, gypenosides LXXXXI–LXXXVII, together with four known compounds from *G*. *pentaphyllum*. All isolated compounds were assessed for their effect on PCSK9 expression in HepG2 cells. PCSK9 expression was measured by ELISA in LPDS-induced HepG2 cells, and cell viability was assessed via MTT assay to exclude cytotoxic compounds. Several compounds that did not show cytotoxicity at 20 μM were selected for further analysis. Gypenosides LXXXII (**30**), LXXXV (**31**), and LXXXVII (**32**) showed PCSK9 inhibitory effects at 10 μM, although LXXXVII (**32**) showed some cytotoxicity. Notably, gypenoside LXXXII (**32**) also exhibited inhibitory activity at 5 μM. Further SAR analysis showed that the side chain at C-17 with the double bond at C-24 and C-25, the hydroxyl group at C-12, the oligosugar at C-20, and the methyl group at C-10 might be essential for inhibitory effects against PCSK9.

Zhang et al. [53] reported 40 compounds, including 6 new triterpenoids, alisolinal A–F, from the rhizome of *Alisma plantago*-*aquatica* in 2022, evaluating the promoted LDL uptake of all isolates in HepG2 cells using the DiI-LDL uptake quantified assay. Among the tested compounds, 17 compounds exhibited significant LDL-uptake-promoting activities, with 9 protostane-type triterpenoids showing strong activity. Among them, alisol A 23-acetate (**33**), alisol A 24-acetate (**34**), 16-oxo-11-anhydroalisol A (**35**), and alisol B 23-acetate (**36**) showed the most potent activity. Zhang et al. also reported the SAR analysis of 40 compounds in relation to LDL uptake. The C-17 spirost protostane-type triterpenoids with the *S* configuration showed higher activity than those with the *R* configuration. In addition, dihydroxylation of C-25 alisol A-type triterpenes and esterification of the hydroxyl group at C-24 in alisol F-type compounds led to enhanced activity. The isolated compounds were also assessed for their inhibitory effects on PCSK9 expression in HepG2 cells. Among them, alisol G (**37**) and alisolinal C (**38**) showed significant inhibition rates of 46% and 58%, respectively. Furthermore, alisol G (**37**) also showed a 55.2% inhibition of PCSK9 protein expression in Western blot analysis. The authors carried out further investigations with alisol G (**37**) and found that at 10 μM it decreased PCSK9 mRNA expression, increased LDLR mRNA expression, and promoted LDL uptake, whereas at 1 μM it showed no significant activity.

Huh et al. [54] isolated and reported acylated saponins, flavonoid glycosides, and (+)-catechin (**39**) from the fruits of *Stewartia koreana*. The authors screened the isolated compounds for their regulatory activity on PCSK9 and LDLR expression, but only (+)-catechin (**39**) exhibited inhibitory effects on PCSK9 mRNA levels at 50 μM without cytotoxicity. Consequently, Huh et al. [54] conducted Western blot analysis with (+)-catechin (**39**), which showed upregulation of LDLR mRNA levels and downregulation of PCSK9 mRNA levels.

Pel et al. [55] reported 22 compounds, including 2 new isocoumarins and a new benzofuran, from the dried roots of *Lysimachia vulgaris*. All isolated compounds were tested for their inhibitory effects on PCSK9 and LDLR mRNA expression. Among them, 8′*Z*,11′*Z*-octadecadienyl-6,8-dihydroxyisocoumarin (**40**) and 5-*O*-methylembelin (**41**) inhibited PCSK9 mRNA expression at 20 μM significantly. The authors further evaluated 8′*Z*,11′*Z*-octadecadienyl-6,8-dihydroxyisocoumarin (**40**) and 5-*O*-methylembelin (**41**) at various concentrations, and the IC_50_ values for PCSK9 mRNA inhibition were 11.9 and 4.9 μM, respectively. Pel et al. also examined the effects of these two compounds on LDLR-related genes, and the results revealed that PCSK9 mRNA expression was downregulated by SREBP2.

**Figure 4:**
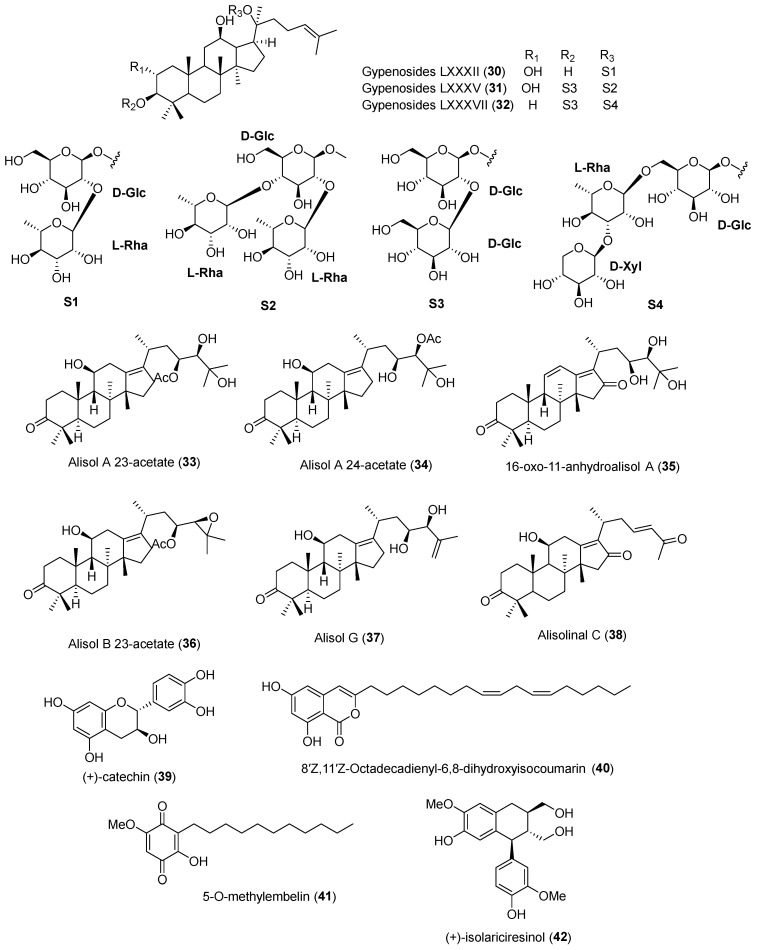
Chemical structures of the naturally occurring PCSK9 inhibitors reported in 2022.

Pel et al. [56] published another paper in 2022, in which they isolated 31 compounds from the roots and rhizomes of *Sophora tonkinensis*. Of these isolated compounds, (+)-isolariciresinol (**42**) showed suppressive effects on PCSK9 protein expression and decreased LDLR protein levels at 10 and 50 μM, as observed in Western blot analysis. Furthermore, (+)-isolariciresinol (**42**) downregulated HNF1α and SREBP mRNA expression, leading to reduced expression of both PCSK9 and LDLR proteins. The chemical structures of the PCSK9 inhibitors reported in 2022 are shown in Figure 4, and the summarized information is presented in Table 3.

### 2.4. The Naturally Occurring PCSK9 Inhibitors Reported in 2023 and 2024

Nhoek [57] isolated and reported 14 new clerodane diterpenoids from the fruits of *Casearia grewiifolia* in 2023. The isolated compounds were evaluated for their regulatory effects on LDLRs, PCSK9, and IDOL expression in HepG2 cells at 20 μM. LDLRs are known to be degraded by PCSK9 and IDOL [58,59]. Among the isolates, grewiifolin C (**43**) showed the strongest inhibition of PCSK9 and IDOL mRNA expression and was chosen for further immunoblot analysis. However, grewiifolin C (**43**) did not exhibit notable inhibitory activity against PCSK9 or IDOL protein expression at 20 and 40 μM.

An et al. [60] isolated 17 compounds, including 3 new acyclic triterpenoids, from the dried seeds of *Alpinia katsumadai*. Among these acyclic triterpenoids, both (3*R*,20*S*)-2,3,20-Trihydroxy-2,6,10,15,19,23-hexamethyl-tetracosa-6,10,14,18,22-pentaene (**44**) and (3*R*,5*S*)-2,3,5-Trihydroxy-2,6,10,15,19,23-hexamethyl-tetracosa-6,10,14,18,22-pentaene (**45**) showed significant inhibitory effects on PCSK9 secretion at 10 and 20 μM, respectively. In addition, compounds **44** and **45** were further evaluated for their effects on PCSK9 and LDLR mRNA expressions. The results showed that compounds **44** and **45** markedly suppressed PCSK9 mRNA levels and promoted LDLR mRNA expression. The authors also tested these two compounds using various concentrations ranging from 0.625 to 10 μM and 1.25 to 20 μM, respectively, and the IC_50_ values were obtained as 2.94 μM for **44** and 15.08 μM for **45**.

In 2024, Hu et al. [61] purified 12 compounds, including 9 amide alkaloids and 3 neolignans, from the aerial part of *Piper hongkongense* and assessed their PCSK9 inhibitory activities in HepG2 cells using the PCSK9 AlphaLISA screening method. In the PCSK9 AlphaLISA screening, hongkongensine C (**46**) and kadsurenone (**47**) showed 38.4% and 52.0% inhibition rates at 5 μM, respectively, and the positive control, berberine, exhibited an inhibition rate of 55.6% at 5 μM. In addition, kadsurenone (**47**) demonstrated dose-dependent inhibition of PCSK9 protein levels in HepG2 cells.

Son et al. [62] reported 5 new compounds, along with 27 known compounds, isolated from the roots of *Cynanchum wilfordii*, and evaluated their inhibitory effects on PCSK9 secretion. The authors conducted a water-soluble tetrazolium-8 (WST-8) assay with 10 μM to assess the cytotoxicity of the isolated compounds in HepG2 cells, and none of the compounds exhibited cytotoxicity. The isolates were then tested for their inhibitory effects on PCSK9 secretion using ELISA screening, in which several compounds showed moderate activity. Among the tested compounds, betulinic acid and (3*S*,8*S*,9*S*,10*R*,13*S*,14*S*,17*S*,22*R*)-24-methylcholesta-5,20,24-trien-3,22-ol (**48**) demonstrated strong inhibitory effects. Since the PCSK9-inhibitory activity of betulinic acid had already been reported [63], the authors selected (3*S*,8*S*,9*S*,10*R*,13*S*,14*S*,17*S*,22*R*)-24-methylcholesta-5,20,24-trien-3,22-ol (**48**) for further investigation. The compound was evaluated for its LDLR and PCSK9 mRNA expression, and the results showed suppressed PCSK9 expression with 2.5 (63%), 5 (27%), and 10 (27%) μM and slightly increased but no significant LDLR mRNA expression. The authors also conducted a Western blot analysis, and the PCSK9 protein levels with this compound were remarkably decreased at 5 and 10 μM. To assess the effect of **48** on transcription factors, qPCR experiments were conducted. Previous studies have identified SREBP1/2 and HNF1α as major regulators of PCSK9 expression [64], and berberine, a known PCSK9 inhibitor, has been reported to downregulate both factors [65]. However, unlike berberine, compound **48** was found to increase the mRNA levels of SREBP 1/2 and HNF1α, suggesting that it regulates PCSK9 via a different pathway. Further analysis revealed that the compound also upregulated the expression of the forkhead box protein O1 (FOXO1) and FOXO3, alternative transcriptional regulators of PCSK9 [66]. In particular, FOXO3 was shown to bind to the PCSK9 promoter and interact with SIRT6, thereby suppressing PCSK9 gene expression through histone H3 deacetylation. Moreover, FOXO3 competitively inhibited HNF1α-mediated upregulation by binding to the HNF1α-binding site on the PCSK9 promoter. These findings indicate that the compound downregulates PCSK9 by increasing FOXO3 levels. Based on these results, Son et al. [62] evaluated the effect of co-treatment with atorvastatin and compound **48**. While administration of 10 μM atorvastatin alone significantly increased PCSK9 mRNA expression, co-treatment with 2.5 μM of (3*S*,8*S*,9*S*,10*R*,13*S*,14*S*,17*S*,22*R*)-24-methylcholesta-5,20,24-trien-3,22-ol (**48**) markedly reduced PCSK9 mRNA levels compared to atorvastatin alone.

In 2024, Wei et al. [67] isolated and reported four new isoquinoline alkaloids from *Hypecoum erectum* and evaluated their PCSK9 inhibition effects. All four isolated compounds and the positive control, PF-06446846, were evaluated for their binding affinity with PCSK9 by surface plasmon resonance (SPR) analysis. Among the tested compounds, hypecotumines C (**51**) and D (**52**) showed moderate affinity strength at 95.1 and 59.9 μM, respectively, against PCSK9, compared to hypecotumines A (**49**) and B (**50**). These results suggest that the methylenedioxy moieties located at C-3′ and C-4′ contribute significantly to the affinity strength. In addition, the authors carried out a Western blot assay to assess the protein levels of PCSK9 and LDLRs, and all isolates showed a decreased expression of PCSK9 and an increased expression of LDLR mRNA. Furthermore, all compounds were tested to identify the binding sites on the PCSK9 protein through molecular docking (PDB ID: 6U3X), and the results exhibited that hypecotumine D (**52**) showed π–cation interactions with ARG-458 and formed a salt bridge with ASP-360, resulting in a binding pattern similar to that of PF-06446846. These interactions were not observed in hypecotumines A–C (**49**–**51**), which may explain the better affinity of hypecotumine D (**52**).

**Figure 5:**
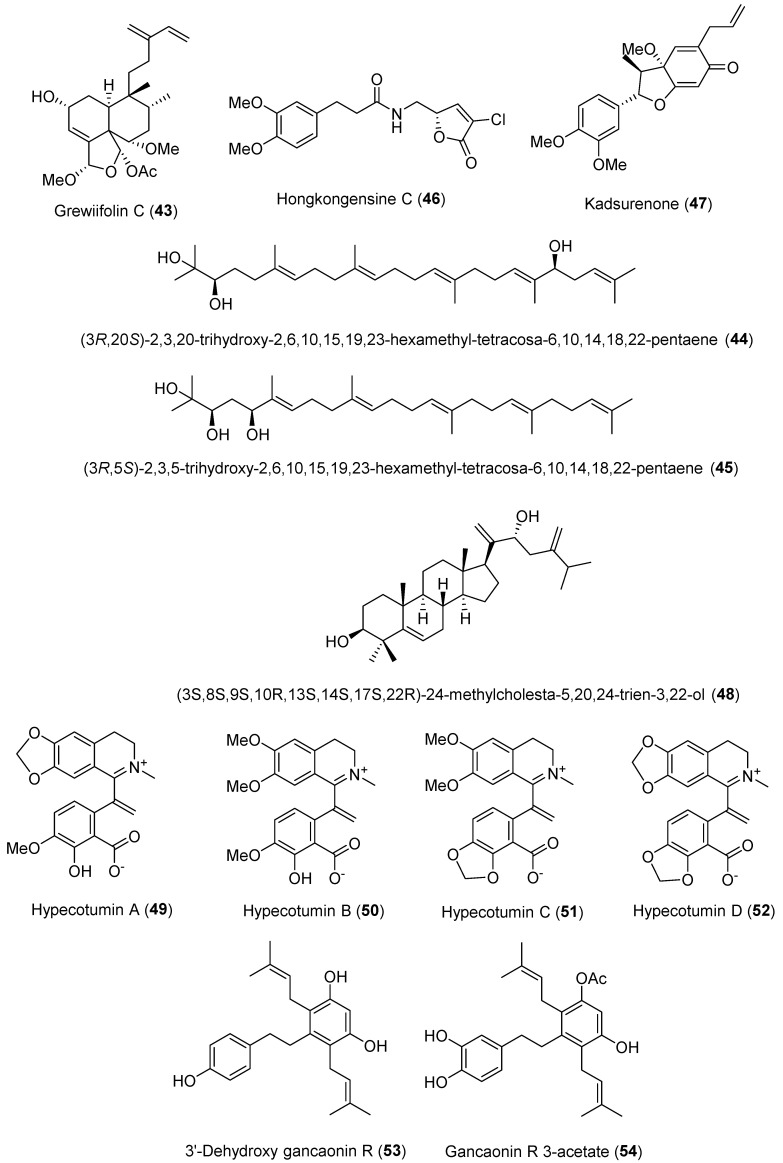
Chemical structures of the naturally occurring PCSK9 inhibitors reported in 2023 and 2024.

Lee et al. [68] confirmed 20 compounds from the whole plants of *Jacobaea vulgaris*. Among the isolated compounds, two stilbene derivatives, 3′-dehydroxy gancaonin R (**53**) and gancaonin R 3-acetate (**54**), significantly inhibited PCSK9 mRNA expression and promoted LDLR mRNA expression at 20 μM. The authors also tested these two compounds (**53** and **54**) at various concentrations ranging from 6.25 to 50 μM and determined their IC_50_ values for PCSK9 protein inhibition to be 16.1 and 20.6 μM, respectively. In addition, the effects of the compounds on LDLRs, PCSK9, and IDOL protein levels were examined, and the results showed that 3′-dehydroxy gancaonin R (**53**) upregulated the mature form of LDLR protein, while gancaonin R 3-acetate (**54**) increased LDLR protein levels and suppressed IDOL protein expression. The chemical structures of the PCSK9 inhibitors reported in 2023 and 2024 are depicted in Figure 5, and the summarized information is presented in Table 4.

### 2.5. The Naturally Occurring PCSK9 Inhibitors Reported in 2025

**Figure 6:**
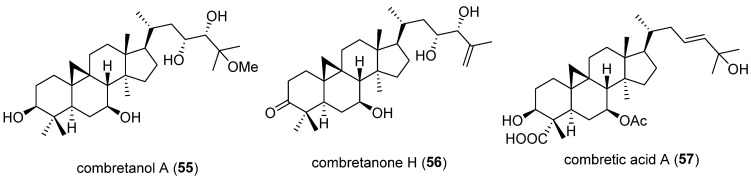
Chemical structures of the naturally occurring PCSK9 inhibitors reported in 2025.

An et al. [69] confirmed a total of 16 cycloartane-type triterpenoids, including 9 new compounds, from the ethanolic extract of *Combretum quadrangulare* twigs and evaluated the isolates for PCSK9 secretion inhibitory activities. All isolated compounds were tested for their inhibitory activity against PCSK9 protein secretion at 20 μM. Among them, combretanol A (**55**), combretanone H (**56**), and combretic acid A (**57**) showed significant inhibition. In addition, a qPCR assay was also conducted using berberine as a positive control alongside the three compounds, revealing suppressed PCSK9 mRNA expression and promoted LDLR mRNA expression. In Western blot analysis, all three compounds reduced PCSK9 protein levels and downregulated mature PCSK9 (65 kDa), while only combretic acid A (**57**) significantly increased LDLR protein levels. Furthermore, to assess their ability to counteract atorvastatin-induced PCSK9 elevation, the three compounds (**55**–**57**) were co-treated with atorvastatin. All three compounds demonstrated inhibitory effects on atorvastatin-induced PCSK9 expression at 10 μM. Of these, combretic acid A (**57**) was selected for further evaluation due to its ability to increase LDLR protein expression. DiI-LDL staining in HepG2 cells confirmed that combretic acid A (**57**) remarkably increased LDL uptake at 10 μM. A pharmacokinetic study of combretic acid A (**57**) was performed in mice via intraperitoneal (IP) injection. It was rapidly absorbed and extensively distributed in the liver, where its concentration remained notably higher than in plasma from 60 to 300 min (T/P ratios > 1). Additionally, the AUC_0-300_ of combretic acid A (**57**) in the liver (60.8 μg min/g tissue) was notably higher than that in plasma AUC (7.62 μg min/mL), indicating that the compound remains and acts primarily in the liver. Given that PCSK9 is predominantly produced and secreted in the liver, these findings suggest that combretic acid A (**57**) has potential as a promising therapeutic candidate for lowering PCSK9 production. The chemical structures of the PCSK9 inhibitors reported in 2025 are shown in Figure 6, and the summarized information is presented in Table 5.

## 3. Methodology

The keywords used in the review were “PCSK9 inhibitors”, “Natural”, and “Plant”, and searches were conducted using the Web of Science, PubMed, Google Scholar, and Scifinder databases. Publications from January 2020 to April 2025 were searched, excluding those focusing solely on extracts or review articles. In addition, previously reported studies on known PCSK9 inhibitors were excluded. The chemical structures were drawn by ChemDraw 23.1.2 software.

## 4. Conclusions and Future Perspectives

Since January 2020, concerted efforts have been made to isolate small molecules with PCSK9 inhibitory activity from natural products, based on research published on PCSK9 inhibitors during this period. As a result, a total of 350 compounds were reported between 2020 and April 2025, among which 57 compounds were confirmed to exhibit inhibitory activity against PCSK9. These active compounds were isolated from 18 plant species with diverse chemical profiles and include 19 isoprenoids, comprising 1 diterpene, 3 sesquiterpenes, and 15 triterpenes, along with 7 triterpenoidal saponins, 4 flavonoids, 9 flavonoid glycosides, 5 alkaloids, 1 isocoumarin, 5 lignans, 1 nucleic acid, 1 phenanthrene glycoside, 1 benzoquinone, 1 selaginellin derivative, and 3 stilbenes.

In addition, these isolated compounds modulate PCSK9 through various mechanisms, such as suppression of mRNA expression, downregulation of transcription regulators (HNF1α, SREBP2, FOXO1, and FOXO3), enhancement of LDLR activity, and inhibition of PCSK9-LDLR binding. Compared to earlier reviews, this study not only provides an updated overview of naturally derived compounds with proven PCSK9 inhibitory activity but also broadens the chemical diversity of PCSK9 inhibitors by introducing compound classes not previously reported. Overall, this review provides up-to-date information on naturally derived compounds with PCSK9 inhibitory activity and serves as a foundation for the development of drug candidates derived from natural products. However, most of the reported articles are limited to in vitro experiments using HepG2 cell lines, where the PCSK9 inhibitory effects were primarily assessed at the mRNA expression level. To further advance these compounds as viable therapeutic candidates, additional studies are needed, including in vivo investigations and/or the integration of RNA sequencing data with in silico approaches to identify potential interaction sites or direct target proteins [70,71]. These efforts are expected to provide deeper insights into the underlying mechanisms of PCSK9 inhibition.

## Figures and Tables

**Table 1:** Summary of PCSK9 inhibitors reported in 2020.

No.	Compound Name	Compound Class	Origin/Source	Study Model	Activity Level	Mechanism	References
**1**	23,24-dihydrocucurbitacin B	Triterpenoid	*Trichosanthes cucumeroides* roots	HepG2 cells		Inhibits PCSK9 and HNF-1α level; increases LDLR and SREBP2 levels	[43]
				HFD-fed hamsters	30 mg/kg; 50% downregulated; 80% and 70% increased		
**2**	Hexanorisocucurtitacin D			HepG2 cells	5 μM; LDL uptake rate of 2.53	Suppresses PCSK9 mRNA; enhances LDLR mRNA	[44]
**3**	Isocucurbitacin D				5 μM; LDL uptake rate of 2.47		[44]
**4**	(+)-8b-*epi*-ampelopsin A	Stilbene	*Chromolaena odorata* aerial parts	HepG2 cells	IC_50_ 20.6 μM	Inhibits PCSK9 mRNA expression	[45]
**5**	5,6,7,4′-tetramethoxyflavanone	Flavonoid			IC_50_ 21.4 μM		
**6**	5,6,7,3′,4′-pentamethoxyflavanone				IC_50_ 31.7 μM		
**7**	Acacetin				IC_50_ 15.0 μM		
**8**	Uridine	Nucleic acid			IC_50_ 13.7 μM		

**Table 2:** Summary of PCSK9 inhibitors reported in 2021.

No.	Compound Name	Compound Class	Origin/Source	Study Model	Activity Level	Mechanism	References
**9**	Plebeic acid A	Sesquiterpene	*Salvia plebeian* roots	HepG2 cells	IC_50_ 24.4 μM	Inhibitory effects on PCSK9 mRNA expression; upregulates LDLR mRNA expression	[47]
**10**	(1*S*,5*S*,8*S*,10*R*)-1-acetoxy-8-methoxy-2-oxoeudesman-3,7(11)-dien-8,12-olide				IC_50_ 25.2 μM		
**11**	Eudebeiolide B				IC_50_ 27.8 μM		
**12**	Gypenoside LXXXIX	Triterpenoidal saponin	Whole herb of *Gynostema pentaphyllum*	HepG2 cells	20 μM	Inhibition against simvastatin-induced PCSK9 expression	[48]
**13**	Gypenoside XC				10 and 20 μM		
**14**	Gypenoside XCI				10 and 20 μM		
**15**	Ginsenoside Rg5				10 and 20 μM		
**16**	Icariside I	Flavonoid glycoside	*Epimedium koreanum* aerial parts	HepG2 cells	10 μM	Inhibits PCSK9 mRNA expression	[49]
**17**	Ikarisoside A					Inhibits PCSK9 mRNA expression; increases LDLR mRNA expression	
**18**	Icariin					Inhibits PCSK9 mRNA expression	
**19**	Anhydroicaritin 3-*O*-β-d-fucopyranosyl(1→2)-rhamnopyranoside-7-*O*-β-d-glucoside						
**20**	Korepimedoside A						
**21**	Epimedokoreanoside I						
**22**	Korepimeoside C						
**23**	Epimedin L						
**24**	Caohuoside B						
**25**	Epimedoicarisoside A						
**26**	Selaginpulvilin U	Selaginellin derivative	*Selaginella tamariscina* roots and rhizophores	HepG2 cells	50 μM	Increases LDLR expression	[50]
**27**	Obovatalin A	Lignan	Dried bark of *Magnolia obovate*	HepG2 cells	IC_50_ 12.0 μM	Inhibitory effects on PCSK9 protein levels and increases LDLR expression	[51]
**28**	Obovatalin B				IC_50_ 45.4 μM		
**29**	Magnolol				IC_50_ 22.9 μM		

**Table 3:** Summary of PCSK9 inhibitors reported in 2022.

No.	Compound Name	Compound Class	Origin/Source	Study Model	Activity Level	Mechanism	References
**30**	Gypenoside LXXXII	Triterpenoidal saponin	Whole herb of *Gynostema pentaphyllum*	HepG2 cells	5, 10 and 20 μM	Inhibition against LPDS-induced PCSK9 expression	[52]
**31**	Gypenoside LXXXV				10 and 20 μM		
**32**	Gypenoside LXXXVII				20 μM		
**33**	Alisol A 23-acetate	Triterpene	*Alisma plantago*-*aquatica* rhizomes	HepG2 cells	10 μM	Inhibits PCSK9 mRNA expression	[53]
**34**	Alisol A 24-acetate	Triterpene					
**35**	16-oxo-11-anhydroalisol A	Triterpene					
**36**	Alisol B 23-acetate	Triterpene					
**37**	Alisol G	Triterpene			58%, 10 μM		
**38**	Alisolinal C	Triterpene			46%, 10 μM		
**39**	(+)-Catechin	Flavonoid	*Stewartia koreana* fruits	HepG2 cells	50 μM	Suppresses PCSK9 protein levels and increases LDLR levels	[54]
**40**	8′*Z*,11′*Z*-octadecadienyl-6,8-dihydroxyisocoumarin	Isocoumarin	*Lysimachia vulgaris* roots	HepG2 cells	IC_50_ 11.9 μM	Inhibits PCSK9 mRNA expression	[55]
**41**	5-*O*-methylembelin	Benzofuran			IC_50_ 4.9 μM		
**42**	(+)-Isolariciresinol	Lignan	*Sophora tonkinensis* rhizomes	HepG2 cells	10 and 50 μM	Downregulates HNF1α and SREBP mRNA expression; reduces expression of PCSK9 and LDLR protein	[56]

LPDS: lipoprotein-deficient serum.

**Table 4:** Summary of PCSK9 inhibitors reported in 2023 and 2024.

No.	Compound Name	Compound Class	Origin/Source	Study Model	Activity Level	Mechanism	References
**43**	Grewiifolin C	Diterpene	*Casearia grewiifolia* fruits	HepG2 cells	20 μM	Inhibits PCSK9 and IDOL mRNA expression	[57]
**44**	(3*R*,20*S*)-2,3,20-trihydroxy-2,6,10,15,19,23-hexamethyl-tetracosa-6,10,14,18,22-pentaene	Acyclic triterpenoid	Dried seeds of *Alpinia katsumadai*	HepG2 cells	IC_50_ 2.94 μM	Inhibition of PCSK9 mRNA expression	[60]
**45**	(3*R*,5*S*)-2,3,5-trihydroxy-2,6,10,15,19,23-hexamethyl-tetracosa-6,10,14,18,22-pentaene				IC_50_ 15.08 μM		
**46**	Hongkongensine C	Amide alkaloid	Aerial part of *Piper hongkongense*	HepG2 cells	5 μM, 38.4%	Inhibitory activity against PCSK9 expression	[61]
**47**	Kadsurenone	Lignan			5 μM, 52.0%		
**48**	(3*S*,8*S*,9*S*,10*R*,13*S*,14*S*,17*S*,22*R*)-24-methylcholesta-5,20,24-trien-3,22-ol	Triterpene	*Cynanchum wilfordii* roots	HepG2 cells	2.5 (63%), 5 (27%), and 10 (27%) μM	Suppresses PCSK9 expression	[63]
**49**	Hypecotumine A	Isoquinoline alkaloids	Whole herb of *Hypecoum erectum*	Affinity with PCSK9 protein by SPR analysis	K_D_ 306.0 μM	Downregulates PCSK9 protein levels; upregulates LDLR protein levels	[67]
**50**	Hypecotumine B				K_D_ 248.0 μM		
**51**	Hypecotumine C				K_D_ 95.1 μM		
**52**	Hypecotumine D				K_D_ 59.9 μM		
**53**	3′-dehydroxy gancaonin R	Stilbenes	Whole herb of *Jacobaea vulgaris*	HepG2 cells	IC_50_ 16.1 μM	Inhibition of PCSK9 mRNA expression; upregulation of LDLR protein levels	[68]
**54**	Gancaonin R 3-acetate				IC_50_ 20.6 μM		

SPR: surface plasmon resonance.

**Table 5:** Summary of PCSK9 inhibitors reported in 2025.

No.	Compound Name	Compound Class	Origin/Source	Study Model	Activity Level	Mechanism	References
**55**	Combretanol A	Triterpenoid	*Combretum quadrangulare* twigs	HepG2 cells	20 μM	Suppresses PCSK9 mRNA expression; promotes LDLR mRNA expression	[69]
**56**	Combretanone H				5 and 10 μM		
**57**	Combretic acid A				2.5, 5, 10, and 20 μM		

## Data Availability

No new data were created or analyzed in this study. Data sharing is not applicable to this article.

## Abbreviations

The following abbreviations are used in this manuscript: AUC, Area under the curve; PCSK9, Proprotein convertase subtilisin/Kexin type 9; SREBP, Sterol regulatory element-binding protein; LDL, Low-density lipoprotein; DiI-LDL, 1,1′-dioctadecyl-3,3,3′,3′-tetramethylindocarbocyanine perchlorate-labeled low-density lipoprotein; HNF-1α, Hepatocyte nuclear factor-1 alpha; FOXO1, Forkhead box protein O1; FOXO3, Forkhead box protein O3; EGCG, Epigallocatechin gallate; mRNA, Messenger RNA; IDOL, Inducible degrader of the low-density lipoprotein receptor; IC_50_, Half-maximal inhibitory concentration; WST-8, Water-soluble tetrazolium 8; IP, Intraperitoneal; T/P ratio, Trough-to-peak ratio.
